# Supplementary material for: Emergency Lung Transplantation after COVID-19: Immunopathological Insights on Two Affected Patients
Source: Cells. 2021 Mar 10;10(3):611. doi: 10.3390/cells10030611 (PMC7999589; doi:10.3390/cells10030611)
Supplement: Supplementary file 1 [file cells-10-00611-s001.pdf]

Emergency lung transplantation after COVID-19: immunopathological insights on two affected patients.

Giorgio A Croci, Valentina Vaira, Daria Trabattoni, Mara Biasin, Luca Valenti, Guido Baselli, Massimo Barberis, Elena Guerini Rocco, Giuliana Gregato, Mara Scandroglio, Evgeny Fominskiy, Alessandro Palleschi, Lorenzo Rosso, Mario Nosotti, Mario Clerici, Stefano Ferrero

Supplementary Information

SI includes 3 Supplementary figures and 4 Supplementary tables

Supplementary Figures

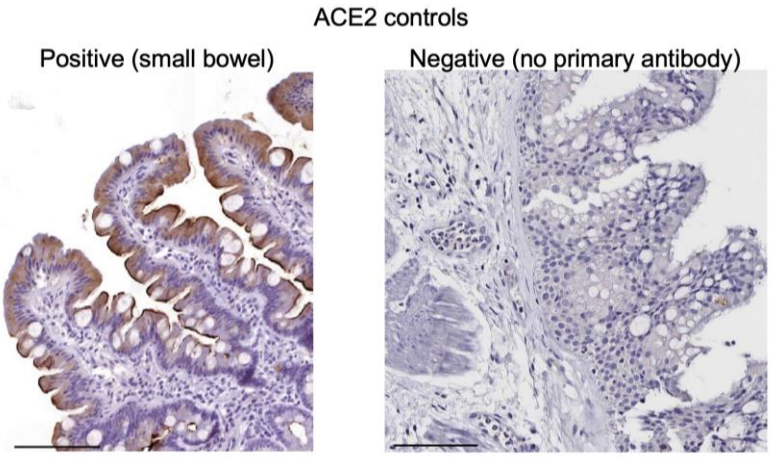

Figure S1. Representative images of a positive (small bowel) and negative (slide incubated with only the secondary antibody) for ACE2 are shown. Scale bars, 100 μm.

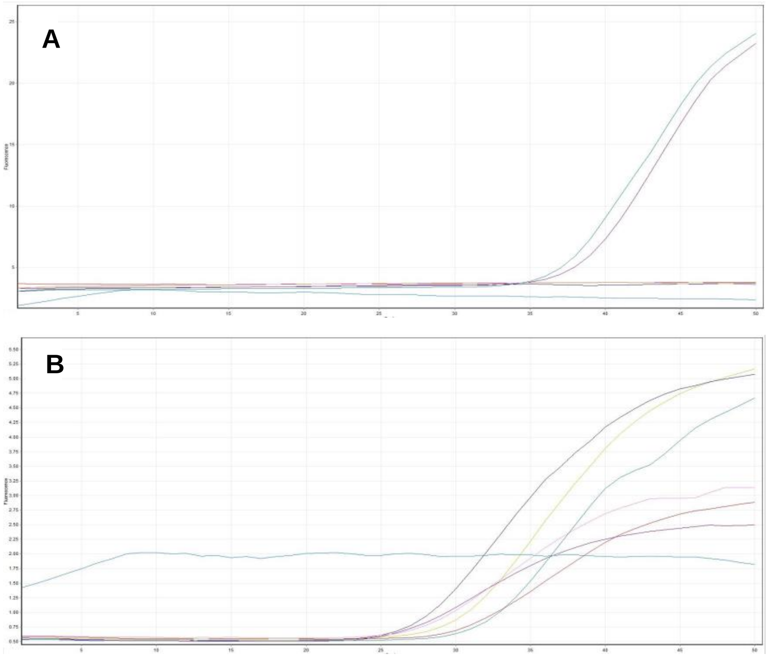

Figure S2. SARS-CoV-2 analysis by real-time RT-PCR. FFPE samples (lung and lymph nodes) from the two COVID-19 LTx patients were analyzed for presence of the viral capside genomic regions together with positive and negative controls. Panel A shows detection of the viral genome in the lymph node from patient #1 and in controls (Ct<45). Amplification curves of the extraction control in all samples and negative reaction control (light blue curve) are showed in panel B.

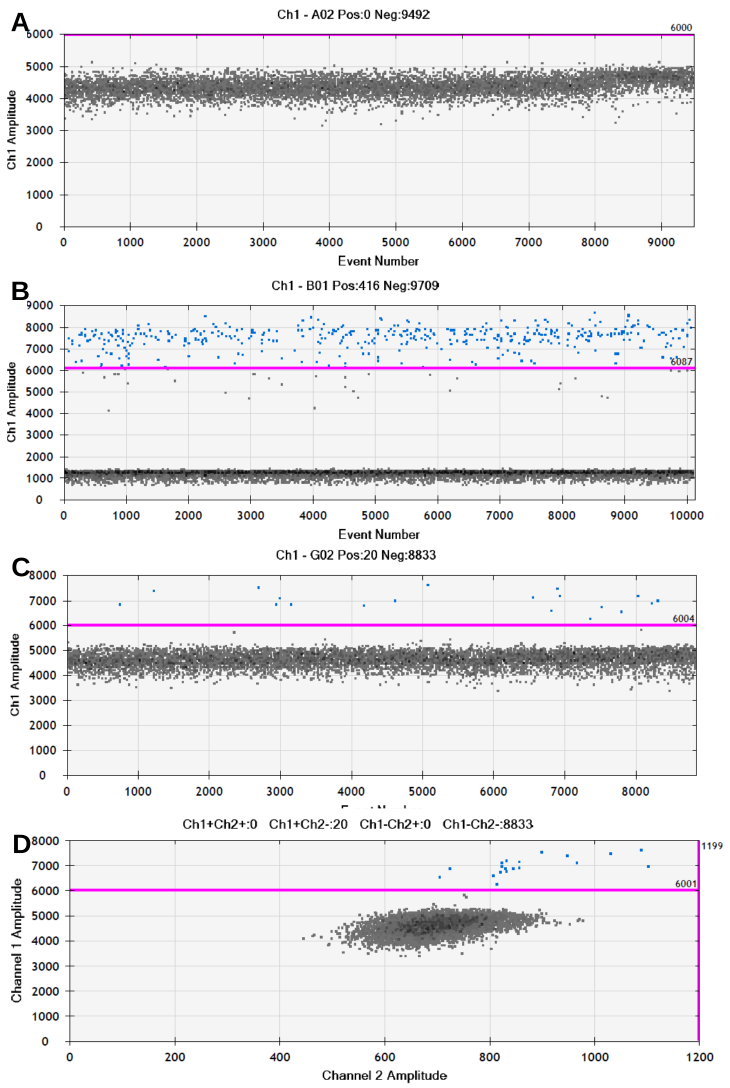

Figure S3. SARS-CoV-2 analysis by ddPCR. The viral genome (region N1) was analyzed by ddPCR in lungs and lymph nodes from the two COVID-19 LTx patients. Scatter Plot for a negative control (A), a positive control (B), the lymph node (C) or the lung samples (D) from patient #1 are showed.

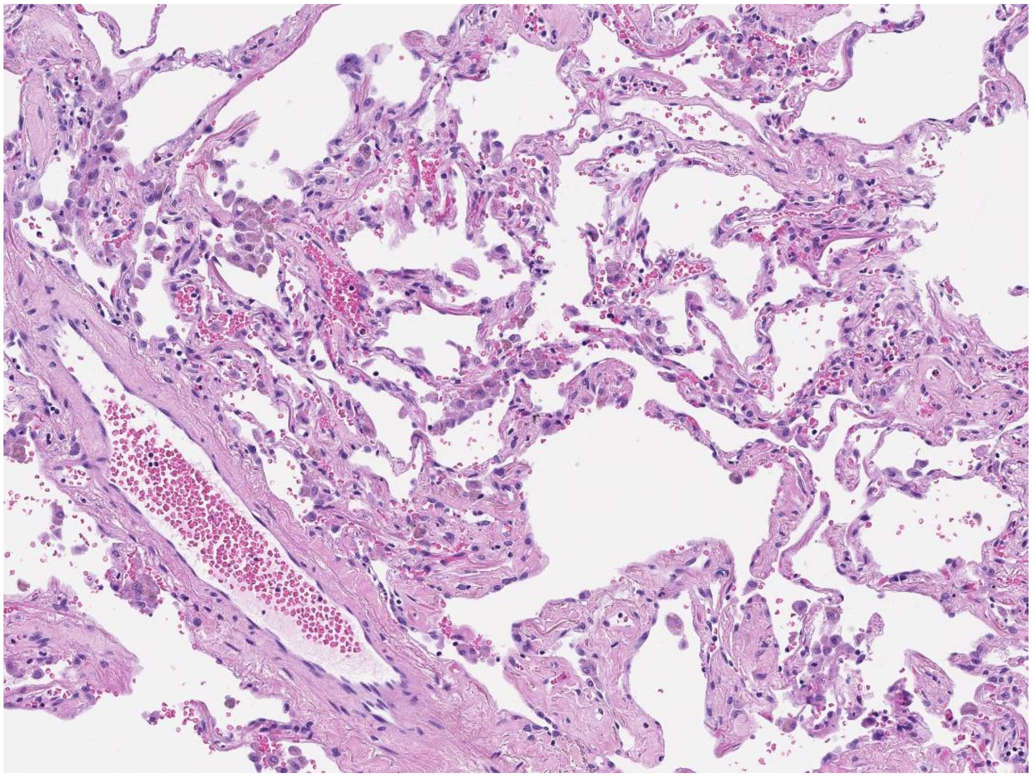

Figure S4. Representative image of a lung from a patient that underwent surgery for non end-stage chronic respiratory diseases. Detail of non-pathologic lung parenchyma. Apart from minor features of blood vessel congestion, secondary to the surgical intervention (the detail corresponds to normal lung from a lobectomy for lung adenocarcinoma), the pulmonary acini are lined by non-descript, type I alveolar epithelia, with no sign of broadening nor remodeling of the interstitial tissue. Inflammatory cells are barely absent, with only scant alveolar macrophages.

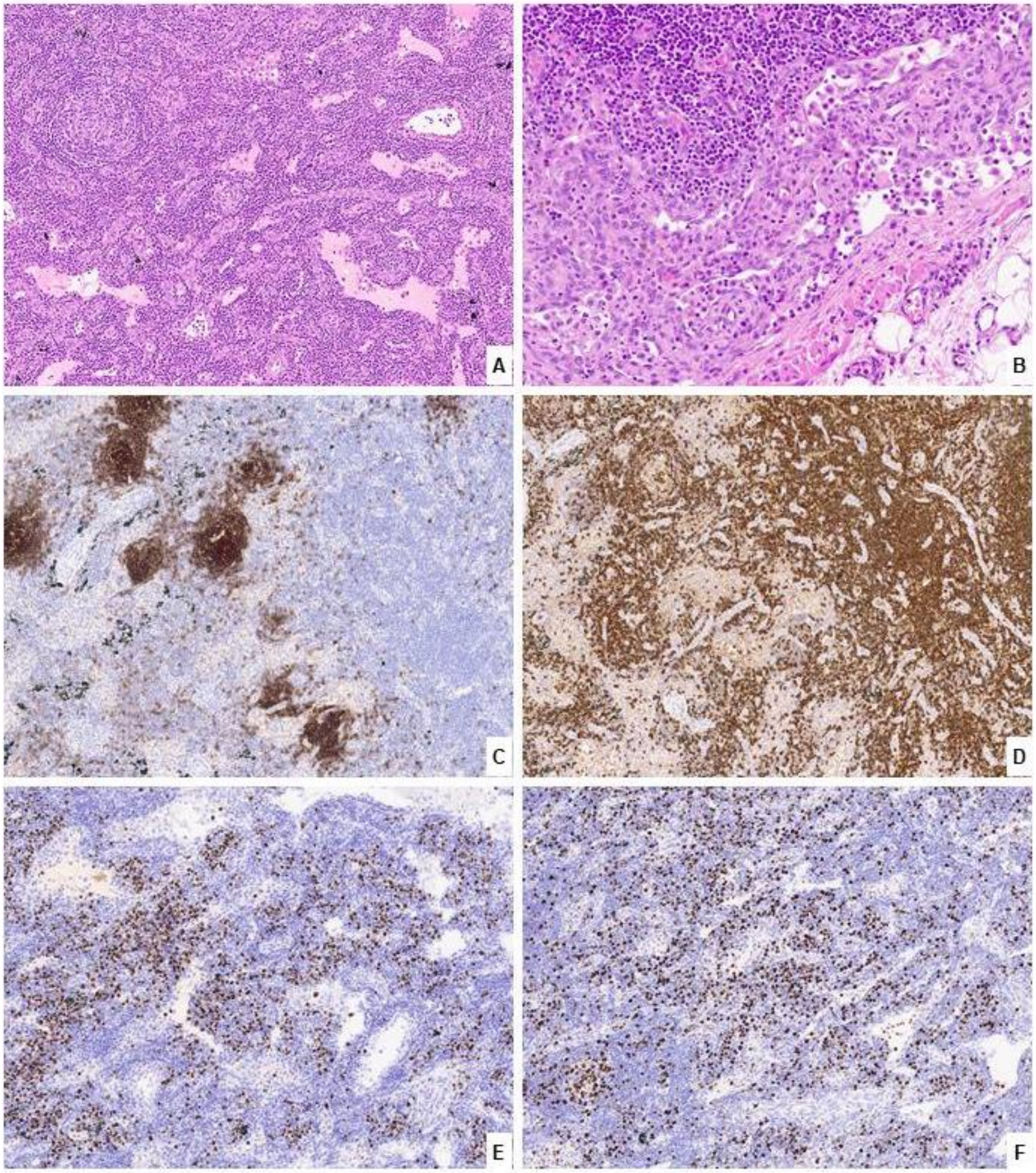

Figure S5. Immunopathological examination of the hilar lymph nodes. Features of nodal hyperplasia with prevalent paracortical expansion, regressed follicular structures, sinus histiocytosis and small vessel proliferation could be detected (A), at times with reactive angioendotheliomatosis pattern (B). The immunophenotype shows that the CD20+ B-cell component (C) is restricted to primary and secondary follicles, and is surrounded by an expanded CD3+ T-cell population (D). MUM1 immunostain (E) reveals a paracortical expansion of immunoblasts, which are the proliferating compartment (i.e. Ki67-positive; F).

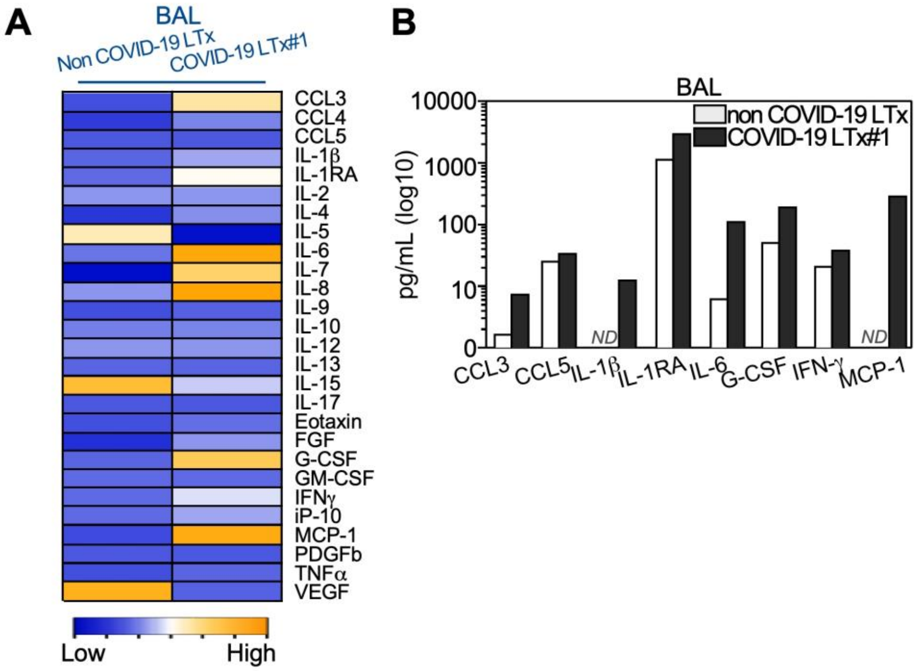

Figure S6. Circulating cytokine and chemokine profile of end-stage COVID-19 lung tissues. The indicated molecule was quantified in the BAL fluids from a non-COVID-19 LTx patient and COVID-19 LTx#1 (A,B). B) Cytokines and chemokines over-represented in both plasma and BAL samples of COVID-19 LTx#1 are shown. *ND*, not determined. Yellow and blue color in the heatmaps show high or low level, respectively (see also Supplemental Table S2 for details).

Supplementary Tables

TABLE S1. Immune genes expression profile of lungs removed from the two COVID-19 LTx patients. Values represent gene relative quantification to endogenous reference genes.

Gene name	COVID LTx#1	COVID LTx#2
CCL2	275,911	197,78
CCL3	14,38751	15,34339
CCL5	16,29876	8,045924
CXCL10	8,282107	0
IL8	85,9534	101,4161
IL1A	2,548341	
IL1B	8,972282	5,80055
IL6	13,64424	2,619603
IL7	4,671958	
IL18	30,84554	40,22962
IL22	3,875601	0,748458
TNF	1,380351	
CSF2	2,548341	
IL10	4,618867	0
IL1RN	67,21248	67,92257
IL6R	73,05243	62,12202
TNFRSF4	2,070527	
TLR4	88,13011	58,0055
TLR3	17,36057	3,929405
TLR7	2,123617	
TLR8	21,39544	19,27279
NOD1	8,813011	0,748458
NOD2	2,707612	
DC-SIGN	19,05946	16,09185
NLRP3	3,291607	1,496916
PYCARD	60,57618	62,49625
CASP1	49,69264	37,04867
CD44	435,5539	408,471
ITGA4	40,08327	22,82797
CH25H	38,17202	17,77588
PPARG	84,51996	85,69844
NR1H3	45,33923	48,08843
HMGCS1	106,1809	76,71695
PTGS2	81,12217	72,60043
ABCA1	176,8973	112,6429
ELOVL6	2,49525	
MX1	76,07858	50,89515
IFITM1	486,149	476,7678
IFITM3	2708,621	2591,91
IFI16	115,047	89,44073
ACE	31,69499	17,40165
ACE2	3,822511	
AGTR1	1,964346	
AGTR2	11,67989	10,85264
TMPRSS2	121,5771	92,24745
CD38	34,82732	35,73887
CD69	15,71477	11,6011
PD-1	1,061809	
PD-L1	56,22276	42,10076

TIM3	11,30826	8,420153
ERAP1	91,31554	64,74162
ERAP2	0,690176	

TABLE S2. Cytokine and chemokinedetection in body fluids from the two COVID-19LTx patients and non-COVID-19 LTx. Data are expressed as pg/mL. *nd*, not detected

PLASMA			BAL			PLASMA-COVID LTx#1			
NAME	COVID LTx#1	COVID LTx#2	NAME	COVID LTx#1	non COVID LTx	NAME	T0	T1	T7
CCL3	10.62	2.52	CCL3	7.23	1.62	CCL3	10.62	8.64	1.65
CCL4	462.08	330.62	CCL4	101.07	3.46	CCL4	462.08	394.73	242.9
CCL5	5950.55	8061.78	CCL5	33.1	24.75	CCL5	5950.55	5637.77	3907.61
IL-1b	78.34	15.92	IL-1b	12.24	nd	IL-1b	78.34	18.2	11.6
IL-1ra	6009.9	1170.47	IL-1ra	2899.84	1111.37	IL-1ra	6009.9	13199.5	153.46
IL-2	8.12	nd	IL-2	nd	nd	IL-2	8.12	nd	nd
IL-4	3.68	2.66	IL-4	0.93	nd	IL-4	3.68	1.97	2.03
IL-5	94.6	89.59	IL-5	73.57	89.59	IL-5	94.6	112.67	nd
IL-6	35.04	6.08	IL-6	108.71	6.08	IL-6	35.04	48.3	3.71
IL-7	25.03	25	IL-7	29.36	0	IL-7	25.03	15.35	20.62
IL-8	34.72	15.21	IL-8	1530.86	13.69	IL-8	34.72	56.58	8.11
IL-9	270.02	318.31	IL-9	19.6	0	IL-9	270.02	254.42	216.19
IL-10	20.78	7.05	IL-10	4.94	4.72	IL-10	20.78	23.18	nd
IL-12	8.13	nd	IL-12	nd	nd	IL-12	8.13	nd	nd
IL-13	31.73	14.42	IL-13	nd	nd	IL-13	31.73	1.32	9.14
IL-15	656.07	263.33	IL-15	508.11	807.91	IL-15	656.07	651.68	nd
IL-17	27.91	27.22	IL-17	nd	nd	IL-17	27.91	22.77	18.09
Eotaxin	31.7	50.71	Eotaxin	5.31	nd	Eotaxin	31.7	28.54	54.18
FGF	55.6	56.38	FGF	18.25	nd	FGF	55.6	54.8	48.26
G-CSF	193.4	34.04	G-CSF	187.76	49.8	G-CSF	193.4	106.79	15.57
GM-CSF	22.74	9.55	GM-CSF	nd	nd	GM-CSF	22.74	10.48	nd
IFN-g	82.5	25.83	IFN-g	37.37	20.44	IFN-g	82.5	169.15	9
IP-10	3157.41	891.75	iP-10	790.59	389.7	iP-10	3157.41	2348.12	319.06
MCP-1	129.78	49.4	MCP-1	282.33	0	MCP-1	129.78	71.27	36.4
PDGF-bb	1480.93	1372.81	PDGF-bb	54.05	46.23	PDGF-bb	1480.93	1252.2	276.08
TNFa	209	167.82	TNFa	16.01	nd	TNFa	209	174.88	103.69
VEGF	429.29	nd	VEGF	nd	779.67	VEGF	429.29	nd	nd

Table S3. Immune genes profile of PBMCs from COVID-19 LTx patients at baseline (mock) or after stimulation with SARS-CoV-2 specific antigens (+SARS). Values represent gene relative quantification to endogenous reference genes.

GENE NAME	mock PBMC-LTx#1	mock PBMC-LTx#2	+SARS PBMC-LTx#1	+SARS PBMC-LTx#2
CCL2	5135.18	742.59	16986.67	4540.34
CCL3	580.34	208.42	2496.38	941.19
CCL5	664.47	71.31	214.14	1115.92
IL8	3590.29	2111.34	14076.6	3420.58
IL6	3.27		124.66	
IL1A	43.6		212.84	13.73
IL1B	430.42	43.87	2047.91	211.79
IL18	6.68		10.11	
IL22	12.14		9.21	14.32
TNF	44.94	40.43	48.38	23.46
IL10	42.14		1.3	0
IL1RN	229.89	383.64	14.01	
IL6R	79.73	62.85	430.15	281.98
TNFRSF4	5.41		55.05	60.3
TLR4	148.12	144.39	4.15	1.89
TLR7	3.56		190.47	47.72
TLR8	75.03	16.22	2.61	3.46
NOD1	2.3		139.48	19.08
NOD2	16.53			1.25
DC-SIGN	13.5		16.13	
NLRP3	9		5.7	3.79
PYCARD	56.97		19.22	0.65
CASP1	71.08	13.96	46.76	37.08
CD44	942.95	721.64	109.43	70.02
ITGA4	82.02	24.69	1604.89	720.05
PPARG	65.34	45.38	61.48	167.87
PTGS2	10.82		149.66	11.57
LXR	73.11	138.35	64.98	1.19
HMGCS1	46.28	7.49	193.7	80.56
ABCA1	166.65	102.52	23.94	46.14
MX1	17.59		316.18	69.33
IFITM1	424.99	54.87	104.71	75.97
IFITM3	64.2	122.4	618.49	771.76
IFI16	131.46	17.73	300.53	87.04
ACE	3.12		154.96	275.72
TMPRSS2	0			27.89
CD38	1.78		7.82	19.88
CD69	66.83		86.07	105.96
PD-L1	17.95		40.88	38.8
TIM3	37.19	5.5	48.69	17.62
ERAP1	139.66	19.85	102.23	252.84
ERAP2	85	92.24745	31.35	89.96

Table S4. Cytokine and chemokine detection in PBMC surnatants from the two COVID-19LTx patients at baseline (mock) or after SARS-CoV-2 antigens stimulation (+SARS) and at different timings after LTx (for patient#1). Data are expressed as pg/mL. *nd*, not detected.

**NAME**	**mock LTx#1**	**+SARS LTx#1**	**mock LTx#2**	**+SARS LTx#2**	**mock LTx#1-T0**	**mock LTx#1-T1**	**mock LTx#1-T7**	**+SARS LTx#1-T0**	**+SARS LTx#1-T1**	**+SARS LTx#1-T7**
CCL3	0,82	10,72	2,49	46,46	0,82		0,47	10,72	21,73	23,15
CCL4	4,5	35,54	9,5	150,91	4,5	1,19	10,33	35,54	69,63	64,47
CCL5	14,99	14,82	20,64	16,84	14,99	7,86	77,73	14,82	8,11	88,77
IL-1b	*nd*	*nd*	*nd*	1,76	*nd*	*nd*	*nd*	*nd*	0,56	0,47
IL-1ra	23,68	25,19	75,44	86,86	23,68	*nd*	16,7	25,19	25,19	25,19
IL-2	*nd*	*nd*	*nd*	2,64	*nd*	*nd*	*nd*	0,4	0,6	0,64
IL-4	*nd*	0,4	0,23	1,6	34,46	31,94	27,67	48,59	32,19	16,89
IL-5	34,46	48,59	33,2	46,31	0,87	1,45	1,01	4,85	9,91	7,41
IL-6	0,87	4,85	1,5	49,85	50,16	9,77	6,39	223,98	250,42	182,23
IL-8	50,16	223,98	343,36	1334,9	4,55	1,64	12,31	5,68	3,22	15,91
IL-9	4,55	5,68	6,53	12,17	1,12	0,91	0,94	1,46	1,35	2,01
IL-10	1,12	1,46	1,44	1,89	158,1	233,77	152,39	219	237,44	182,88
IL-15	158,1	219	158,1	164,02	*nd*	*nd*	*nd*	*nd*	3,22	3,54
IL-17	*nd*	*nd*	*nd*	8,64	*nd*	*nd*	0,46	0,34	*nd*	0,45
FGF	*nd*	9,4	5,19	25,63	*nd*	*nd*	*nd*	9,4	13,1	12,06
G-CSF	33,81	102,36	57,36	263,02	33,81	13,76	31,91	102,36	125,55	124,84
IFN-g	5,24	7,82	5,05	11,41	5,24	3,6	4,73	7,82	6,33	5,11
IP-10	*nd*	*nd*	*nd*	6,59	*nd*	*nd*	6,28		6,28	*nd*
MCP-1	5,34	6,5	16,57	23,92	5,34	3,93	3,64	6,5	7,88	10,41
PDGF-bb	17,3	16,05	20,96	36,49	17,3	10,9	12,87	16,05	12,87	26,87
TNFa	4,74	19,01	8,12	186,09	4,74	*nd*	7,83	19,01	31,36	43,43
VEGF	294,79	269,33	284,6	248,41	294,79	230,9	285,92	269,33	184,78	216,96
